# Supplementary material for: A Novel Biomarker for Acute Kidney Injury, Vanin-1, for Obstructive Nephropathy: A Prospective Cohort Pilot Study
Source: Int J Mol Sci. 2019 Feb 19;20(4):899. doi: 10.3390/ijms20040899 (PMC6412925; doi:10.3390/ijms20040899)
Supplement: Supplementary file 1 [file ijms-20-00899-s001.pdf]

Supplementary Materials

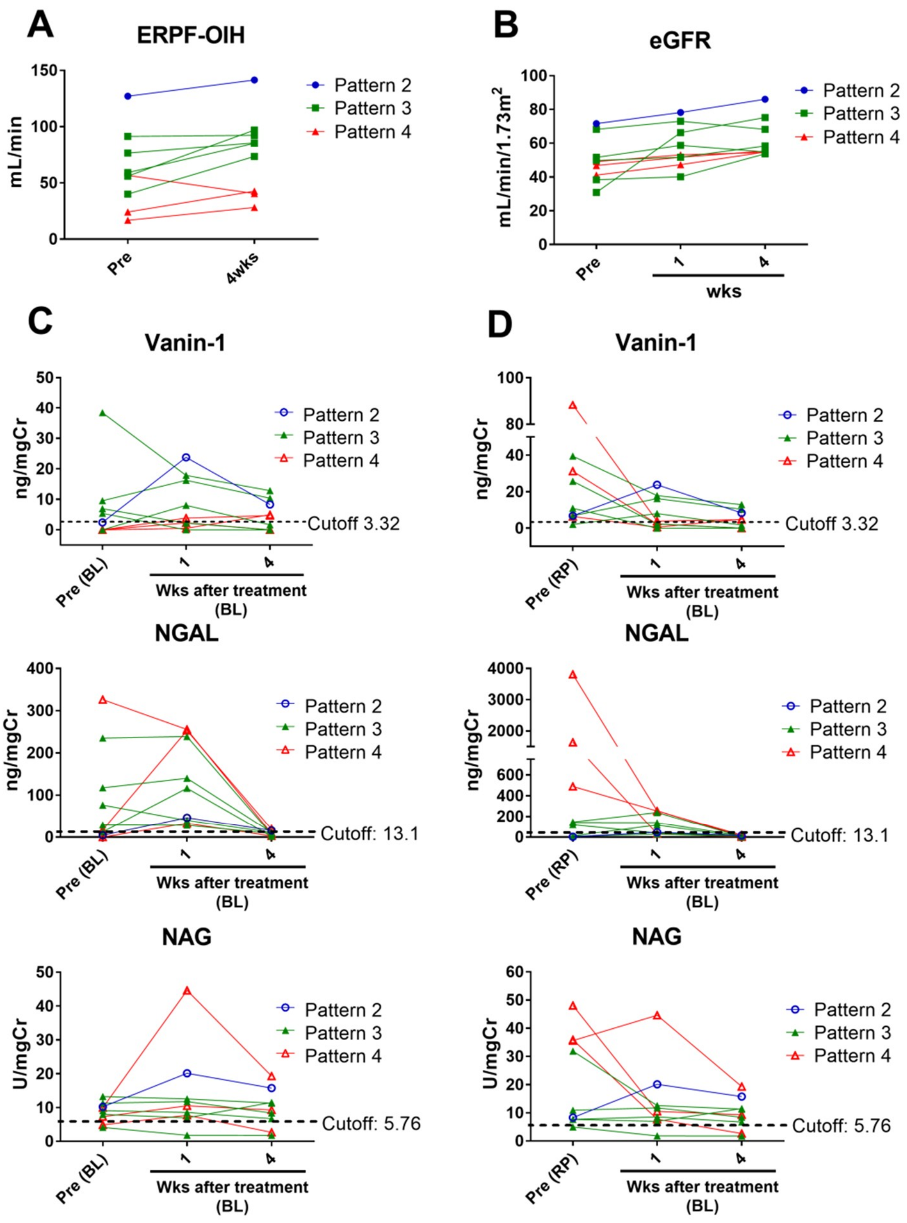

Figure S1. Changes in renal function and each AKI biomarker before and after intervention in each case of extrinsic UUTO. (**A, B**) Changes in renal functions, as assessed by ERPF-OIH (**A**) and eGFR (**B**), before and after intervention in each individual case of extrinsic UUTO (**C, D**). Changes in each AKI biomarker before and after intervention in each individual case of extrinsic UUTO. The levels of each bladder (**C**) and renal pelvic biomarker (**D**) before intervention were compared to those of each bladder biomarker after intervention. The blue circles and line, the green triangles and line, and the red triangles and line indicate pattern 2, pattern 3, and pattern 4 UUTO, respectively. Cut-off for each biomarker in RP urine was also shown.

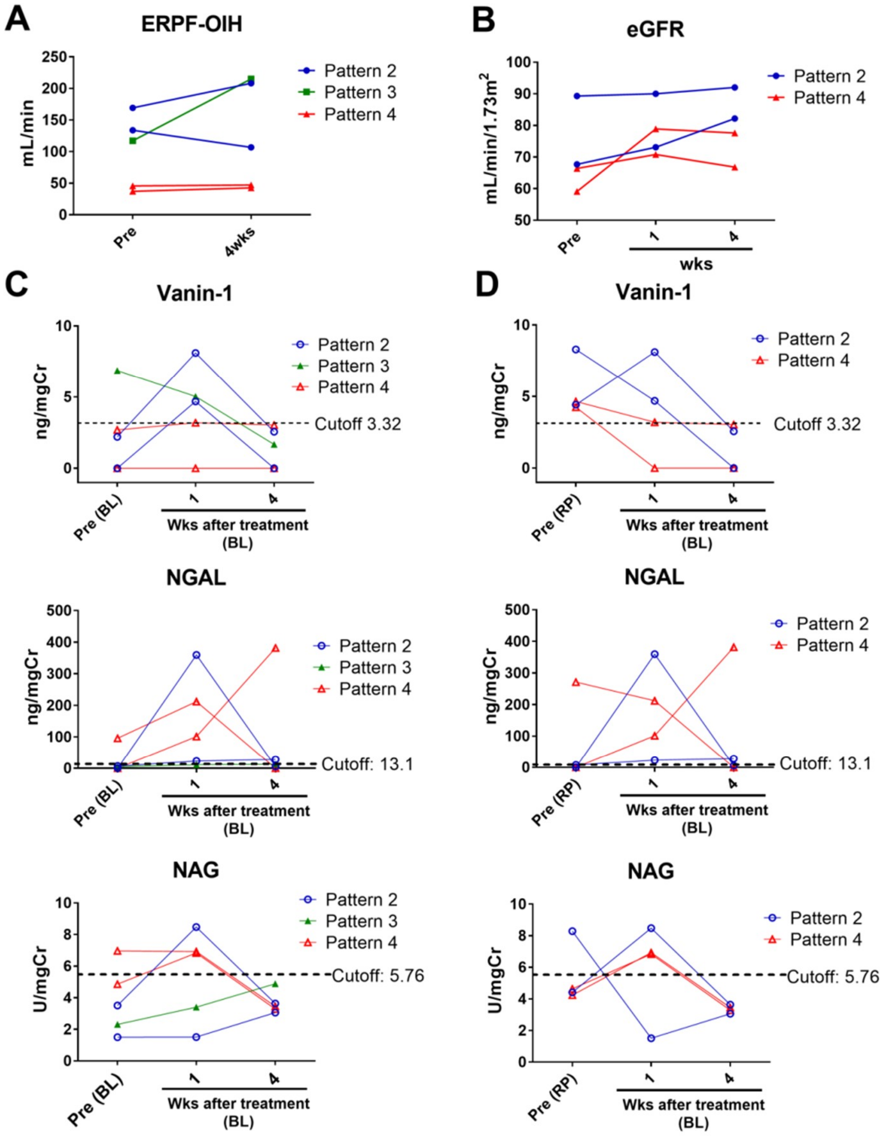

Figure S2. Changes in renal function and each AKI biomarker before and after intervention in each case of intrinsic UUTO. (**A, B**) Changes in renal functions, as assessed by ERPF-OIH (**A**) and eGFR (**B**), before and after intervention in each individual case of intrinsic UUTO (**C, D**). Changes in each AKI biomarker before and after intervention in each individual case of intrinsic UUTO. The levels of each bladder (**C**) and renal pelvic biomarker (**D**) before intervention were compared to those of each bladder biomarker after intervention. The blue circles and line, the green triangles and line, and the red triangles and line indicate pattern 2, pattern 3, and pattern 4 UUTO, respectively. Cut-off for each biomarker in RP urine was also shown.

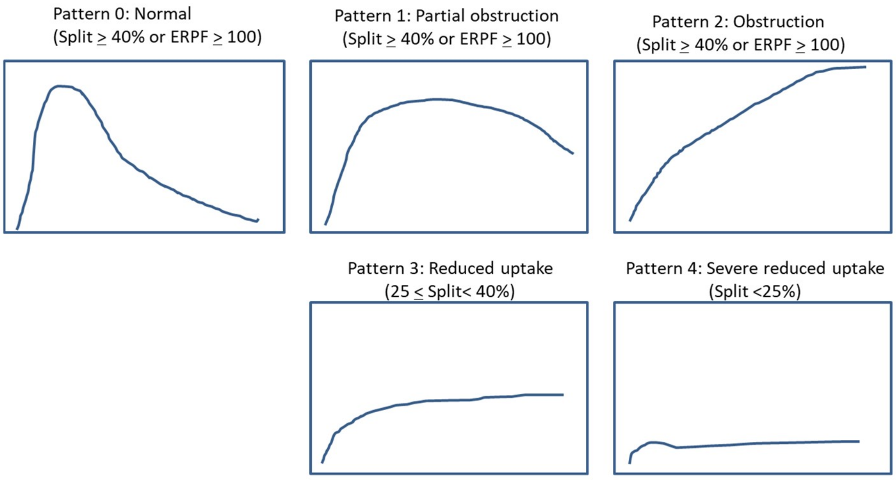

Figure S3. Categorization of hydronephrosis. Hydronephrosis cases were categorized into five UUTO patterns according to the following criteria: pattern 0 (normal: no delayed peak or clearance; normalized ERPF-OIH ≥ 100 mL/min or split ≥ 40%); pattern 1 (partial obstruction: delayed peak or clearance with a downward slope curve and normalized ERPF-OIH ≥ 100 mL/min or split ≥ 40%); pattern 2 (obstruction: consistent upslope curve without peak and normalized ERPF-OIH ≥ 100 mL/min or split ≥ 40%); pattern 3 (reduced uptake: 25%—40% split); and pattern 4 (severe reduced uptake: split < 25%).

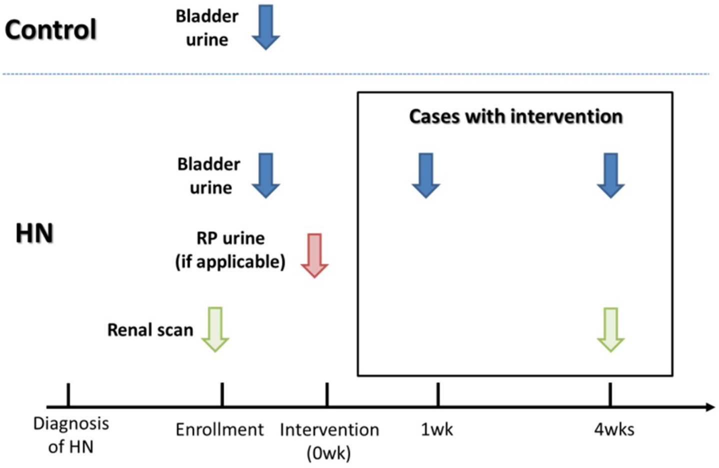

Figure S4. The timing of sample collection and imaging. In the control group, bladder urine samples were collected once after enrollment. In the HN group, they were collected after enrollment and samples of RP urine were collected if applicable. The first MAG3 renal scan was also performed. In cases that underwent interventions for their UUTO, bladder urine samples were collected at 1 week and 4 weeks after the intervention. At 4 weeks after intervention, a second MAG3 renal scan was performed. The blue dotted line separates the timing of sample collection between the control and HN groups.

Table S1. The levels of each biomarker in the control and the hydronephrosis group.

Biomarkers	Unit	Control	HN		
		N = 21	Extrinsic *N* = 16	Intrinsic *N* = 12	Both *N* = 28
**Serum markers**					
**Creatinine**	mg/dL	0.893 ± 0.160	0.986 ± 0.372	0.850 ± 0.302	0.931 ± 0.346

**eGFR**	ml/min/1.73 m^2	68.8 ± 14.0	55.8 ± 17.1		73.1 ± 20.4		62.8 ± 20.1	
**Urine markers**		**BL *N* = 21**	**BL *N* = 16**	**RP *N* = 11**	**BL *N* = 12**	**RP *N* = 4**	**BL *N* = 28**	**RP *N* = 15**
**Vanin-1**	ng/mgCre	0.646 ± 1.117	6.73 ± 9.86	20.5 ± 25.9	2.90 ± 4.55	5.40 ± 1.94	5.09 ± 8.14	16.4 ± 22.9
**KIM-1**	ng/mgCre	0.851 ± 0.973	3.23 ± 5.91	24.62 ± 40	1.15 ± 1.98	0.91 ± 0.428	2.34 ± 4.70	18.3 ± 35.5
**NGAL**	ng/mgCre	8.04 ± 17.3	118 ± 205	583 ± 1170	39.5 ± 84.3	69.9 ± 135	84.2 ± 167	446 ± 1020
**NAG**	U/mgCre	2.90 ± 1.55	8.26 ± 5.40	19.5 ± 15.4	5.86 ± 6.25	4.12 ± 2.05	7.23 ± 5.79	15.4 ± 14.8

All data are shown by mean ± SD. BL, bladder; RP, renal pelvic.
